# Finite Element Study on the Impact Resistance of Laminated and Textile Composites

**DOI:** 10.3390/polym11111798

**Published:** 2019-11-01

**Authors:** Jun Xing, Chunlin Du, Xin He, Zhenqiang Zhao, Chao Zhang, Yulong Li

**Affiliations:** 1 Department of Aeronautical Structure Engineering, Northwestern Polytechnical University, Xi’an 710072, Shaanxi, China; xingjun_acc@caac.gov.cn (J.X.); chunlind@mail.nwpu.edu.cn (C.D.); liyulong@nwpu.edu.cn (Y.L.); 2 Airworthiness Certification Center, Civil Aviation Administration of China, Beijing 100102, China; hexin_acc@caac.gov.cn; 3 Shaanxi Key Laboratory of Impact Dynamics and its Engineering Applications, Xi’an 710072, Shaanxi, China; 4 School of Science, Harbin Institute of Technology, Shenzhen 518055, Guangdong, China

**Keywords:** composite laminates, textile composites, impact damage, numerical simulation, ballistic threshold

## Abstract

The impact resistance of fiber-reinforced polymer composites is a critical concern for structure design in aerospace applications. In this work, experiments were conducted to evaluate the impact performance of four types of composite panels, using a gas-gun test system. Computational efficient finite element models were developed to model the high-speed ballistic impact behavior of laminate and textile composites. The models were first validated by comparing the critical impact threshold and the failure patterns against experimental results. The damage progression and energy evolution behavior were combined to analyze the impact failure process of the composite panels. Numerical parametric studies were designed to investigate the sensitivity of impact resistance against impact attitude, including impact deflection angles and projectile deflection angles, which provide a comprehensive understanding of the damage tolerance of the composite panels. The numerical results elaborate the different impact resistances for laminate and textile composites and their different sensitivities to deflection angles.

## 1. Introduction

Carbon fiber reinforced polymer (CFRP) composites have demonstrated their excellence in reducing the weight of aircraft and hence enhancing economic efficiency, and is increasingly used in aero engines. Typical examples include the application of composites in fan blades and fan case systems of aero engines. The international aviation regulatory organizations have very strict requirements on the impact resistance of fan blades and fan containment systems, in order to guarantee the safety of passengers and crew. Specifically, the engine fan case must demonstrate, experimentally, its capability to contain a failed rotating blade, which can be initiated by fatigue failure, bird strike, or damage from some other foreign object. A full-scale blade-out test involves high-energy and high-speed interactions of engine components, which is a very complicated and expensive problem. Thus, it’s necessary to develop better analysis techniques by introducing laboratory-scale impact tests and virtual tests.

To evaluate the impact resistance of composite materials, Roberts et al. [1] conducted a series of ballistic impact tests on flat and half-ring panels of five different kinds of composites. It was concluded that the ballistic impact limits of composite flat panels can approach that of metals, and the introducing of transverse fiber reinforcement can enhance delamination resistance. Experimentally, Roberts et al. [2] also compared the impact resistance of composites panels made from different types of fabrics and concluded that triaxially braided composites can resist crack initiation and propagation as well as the formation of large delamination between layers during impact loading. 

Ulven et al. [3] investigated the influence of projectile shape on the ballistic limit of carbon/epoxy laminates under high-velocity impact. Yang et al. [4] investigated the high-speed impact failure mechanism and energy absorption feature of 3D braided composites with the assistance of a 3D digital image correlation (DIC) technique for strain-field measurement. Vanderklok et al. [5] investigated the ballistic impact resistance of glass/epoxy composites with a number of layers and identified that a 6-ply (3.9 ± 0.3 mm) panel presented 12% more efficiency in energy absorption than that of a 10-ply (6.19 ± 0.1 mm). Liu et al. [6] studied the containment capability of a 2D triaxially braided composite casing using a spin tester and identified that fiber shear fracture is the main failure mode on the inner wall, while fiber tensile fracture and delamination failure are the main failure modes for the outer wall. Liu et al. [7] conducted a short-beam shear test on aged unidirectional CFRP specimens to evaluate the degradation of interfacial properties and investigage the effect of hygrothermal aging on the ballistic limit of composites. Santos et al. [8] studied the effect of inclined holes on the impact strength of carbon-fiber-reinforced composites, through testing plates with and without holes under impact. The results show that it is crucial to model matrix cracking to achieve a good prediction of delaminated areas.

Rosso et al. [9] performed ballistic impact tests on micro-braid reinforced composites and studied the effect of architecture, braid angle, and different fiber reinforcement. García-Morenoet et al. [10] prepared CFRP laminates after long-term thermal aging treatments and conducted drop-weight impact tests to determine the impact response of the different composite laminates. The result shows that both aging temperature and period are critical design parameters that affect the in-service performance of polymeric–matrix composite structures, especially when the aging temperature is above the glass transition temperature. Pereira et al. [11] quantified the effect of hygrothermal aging on the impact resistance of braided composite panels and found that there is minor reduction in impact performance after 344 cycles of aging.

Impact experiments are mainly used to evaluate impact performance, quantify the impact threshold, and validate the structure design. However, the time and economically costly nature of experiments making numerical simulation the primary tool for the anti-impact design of composite structures. The assessment of numerical models is based on their capabilities in modeling the impact failure process and predicting the impact threshold under various impact conditions. Lee et al. [12,13] proposed a quasi-static model to characterize the penetration process and predict the ballistic limit of composite laminates. Sun et al. [14] employed a structural constitutive model in conjunction with a special two-node ring element based on the Mindlin thick plate theory to model the damage process of composite laminates during static and dynamic penetration. These works predicted well the residual velocities of projectiles after penetrating across or rebounding by the composite panels. Furthermore, to visualize the progressive failure process of plain-woven composite, the ply-level FE model is generally used which considering the fabric-reinforced ply as an orthotropic homogeneous material, with potential capability to sustain plastic deformation and stiffness degradation [15]. In this way, a simple damage mechanics-based maximum energy dissipation approach was presented by Iannucci et al. [16] and implemented into an explicit dynamic FE code, DYNA3D, to predict the impact failure behavior of thin woven composites. Ma et al. [17] presented an efficient methodology to simulate ballistic impact on woven composites and investigated the effect of strain rates on target and projectile deformation.

Muflahi et al. [18] evaluated the capabilities of different element formulations and cohesive fracture models for delamination prediction of thin composite structures, using the commercial software LS-DYNA. Nilakantan [19] conducted the virtual ballistic impact testing of Kevlar soft armor in LS-DYNA and presented a fully validated and predictive probabilistic penetration modeling of a woven fabric through utilizing a finite element model with individually modeled yarns. However, for braided composites with more complicated fabrics architecture, the homogeneous ply-based modeling approach may lose the heterogeneous characteristics of the material. Barauskas et al. [20] proposed a FE model for the ballistic impact of a multi-layer aramid textile package structure in LS-DYNA, which modeled the yarns using thin shell elements and showed good correlation against experiments. Kim [21] performed impact tests, and examined the damage and compressive-after-impact behavior of stitched composite panels with different stitching densities and patterns. Gu et al. [22] implemented the fiber inclination model with the FE code in LS-DYNA to simulate the ballistic penetration process of the 3D braided composite. Tan et al. [23] developed a macro-scale finite element model for impact failure simulation of triaxially braided composites, and numerically investigated the influence of braiding angles on the impact resistance and failure behavior of the composite panel. Similarly, Liu et al. [24] built a macro-scale finite element model for a triaxially braided composite, efficiently simulated the fan-blade-out impact performance of a composite casing structure, and investigated the effect of impact velocity and structure thickness on the damage tolerance of the casing structure. Omar et al. [25] illustrated the different failure behaviors between hollow-core fiber-reinforced polymer columns under dynamic impact conditions. The study was conducted through extensive finite element impact analyses using LS-DYNA software. Zhao [26] proposed a multi-scale modeling framework that can effectively capture the impact failure behavior of a triaxially braided composite. These studies aim to pursue efficient and accurate methods in modeling the impact failure process of braided composites with consideration of the braided architecture; but in realistic engineering applications, the computation efficiency is improved at the expense of accuracy. 

Overall, most of the existing literature studies mainly investigate the impact failure behavior of a single type of composite structure. Till now, there is insufficient understanding of the variation of failure behavior for laminated and textile composites. Thus, in this work, numerical comparison studies were conducted to investigate the variation of impact properties and failure behavior for three types of composite panels (laminated, woven, and triaxially braided composites). Experiments are conducted to validate the numerical models. The paper is organized as follows: Section 2 introduces materials and experiments; Section 3 introduces the finite element model; Section 4 presents model validation and detailed numerical comparison studies for the impact failure behavior of the three different composite panels; and Section 5 summarizes the main findings and conclusions.

## 2. Materials and Experiments

**Figure 1:**
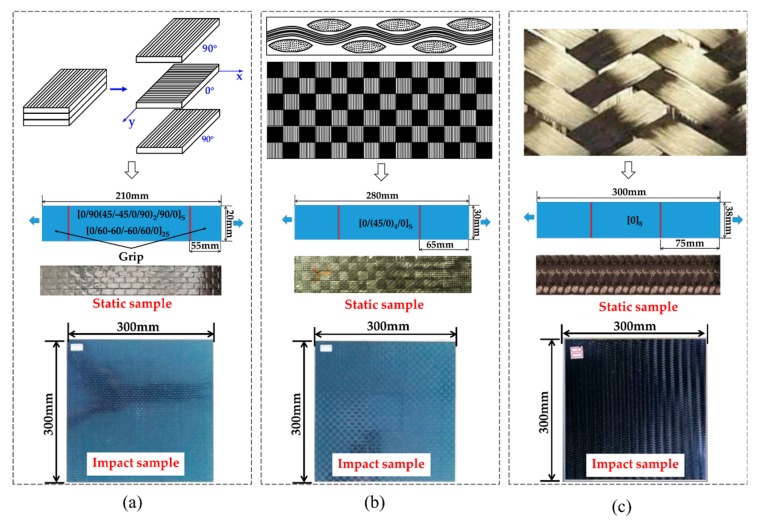
Scheme of architecture features and specimen images for laminate (**a**), woven (**b**) and 2DTBC [21] (**c**) composites.

In order to examine the impact performance of different composites, a series of ballistic experiments were conducted for three kinds of composite panels, including laminated composites, plain-weave woven composites, and two-dimensional triaxially braided composites, of which the laminates were designed to have two different stacking sequences to be comparable with the braided and woven composite panels. A toughened epoxy resin, the 3266-epoxy resin was injected into the T700 carbon fabrics using resin transfer molding (RTM) technology to form the 2DTBC and angle-ply composite panels. During the RTM process, the fabrics are loaded into a heated, rigid, closed mold and the epoxy resin is injected under a pressure of 1 MPa. The cure profile is 3 hours at a maximum temperature of 130 °C. In addition, the plain-woven composite panel is formed by the vacuum-infusion method. All of the composite materials used in this study were provided by Sinoma Science & Technology Co. (Nanjing, China). Figure 1 shows the architecture features, images of static and impact specimens for the three types of composite panels. Table 1 summarizes the detailed geometry information and layup design of the composite panel specimens. All of the composite panels are made from T700 carbon fiber and 3266 epoxy resin, with the same in-plane size of 300 × 300 mm^2^.

To provide fundamental mechanical constants for the impact simulation, quasi-static tensile and compressive characterization tests were designed and conducted for the plain-woven and triaxially braided composite specimens, according to ASTM standards D3039 [27] and D3410 [28]. The long coupon specimens were cut from composite panels, the same as those used for impact tests, using a high-pressure water-jet. A INSTRON 8803 hydraulic testing machine was utilized to load the specimens, and the elongation was obtained by virtue of the electronic extensometer. The quasi-static experimental results of woven and braided composites, including modulus and strength properties, were utilized directly as input parameters for the impact model, as listed in Section 3.2. For the laminated composites, due to the unavailable of sufficient specimens, mature micromechanical theories, including Huang’s model [29] and the Chamis model [30], were employed to determine their basic mechanical constants.

For the ballistic impact experiments, a single-stage gas-gun system was used to accelerate plate-shaped projectiles (50 mm) to velocities in the range of 80~130 m/s. Two high-speed cameras were used to monitor the attitude of the projectiles and to measure the impact velocity. Besides that, laser speedometers were introduced to detect the projectiles, trigger the cameras, and measure the impact velocity of the projectiles. The velocity results from the laser speedometer and the high-speed cameras show differences within 5%, suggesting the reliability of both methods. In this work, the velocity data of the high-speed cameras are used as a reference.

For each type of composite panel, the critical penetration velocity can be defined within a certain interval based on the ballistic impact results. The lower limit of the velocity interval is determined by the maximum impact velocity of the projectile for the cases where the composite panels are not penetrated, and the upper limit is defined as the minimum impact velocity when the projectile penetrates totally into the composite panel. The experimental results for the critical penetration velocity of the four types of composite panels are summarized in Section 4.1 and will be adopted to validate the numerical FE model.

## 3. Numerical Model for Impact Simulation

The impact failure behavior of composite structures varies significantly with the change of impact conditions and can be difficult to investigate through experimental studies only. Thus, numerical models are usually introduced to comprehensively study the impact performance of a composite structure. For comparison purposes, this work uses homogeneous FE models to simulate the impact failure of the studied composite panels, which ignores the fabric’s geometry features and focuses mainly on the effect of effective properties.

### 3.1. Subsection Finite Element Model

**Figure 2:**
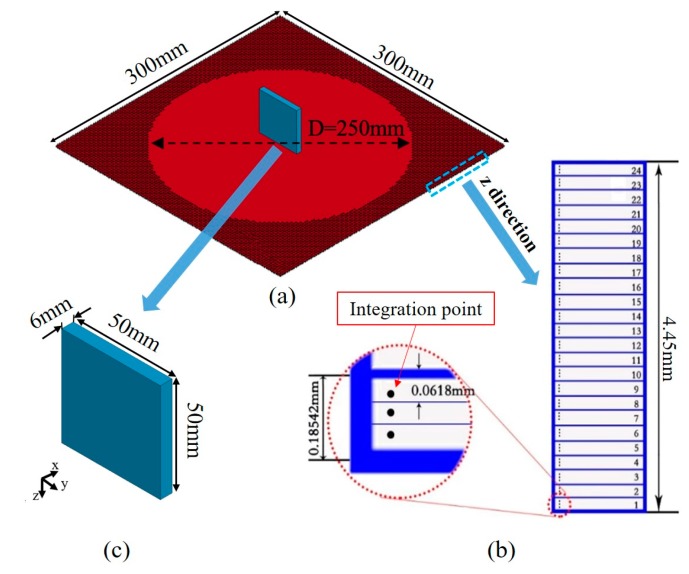
Impact model of the composite panel, (**a**) composite panel and boundary conditions, (**b**) TC4 projectile, (**c**) integration theory of the shell element.

The macro-scale homogenized finite element models for the high-speed impact tests are developed in a commercial software, LS-DYNA. As shown in Figure 2, the model dimension of the metallic (titanium alloy TC4) projectile is 50 × 50 × 6 mm^3^, which is modeled as elastic material for simplification, with basic mechanical parameters. Mass density is 4.4 × 10^−9^ ton/mm^3^, Young’s modulus is 1.1 × 10^5^ MPa, Poisson’s ratio is 0.31.

The model size of each composite panel is listed in Figure 2. The shell element is used to model the composite panel in order to guarantee computational efficiency, with multiple integration points representing the lay-up and thickness of the panel [18], as shown in Figure 2. Detailed information of the FE model for each kind of panel are summarized in Table 1, the two kinds of angle-ply laminates are named as Laminate45 and Laminate60 according to their lay-up sequence, respectively.

The element size is determined to be 3 × 3 mm based on convergence analysis, resulting in a total of 10000 shell elements for a square plate with a size of 300 × 300 mm^2^. For the boundary condition, the four edges of the panel are fully fixed to prevent rigid motion of the panel during the impact. In addition, the center area is out of constraint for a circular area with diameter of 250 mm, while the out-of-plane displacement of the remaining region is constrained. Initial velocity is assigned to the projectile to initiate the impact load. Additionally, automatic surface-to-surface contact is defined between the metallic projectile and the composite panel to model the impact process.

### 3.2. Material Model for the Composites

The studied laminate and textile composites are all considered as orthotropic materials, which can be effectively modeled with the incorporation of proper damage initiation criteria and damage evolution law. In this work, the material model MAT54 implemented in LS-DYNA is used to model all three composite laminates (the actual samples are shown in Figure 1). MAT54 embeds the Chang–Chang [31] failure criterion and a simple stiffness degradation law, as given in Table 2. In Table 2, *X_c_* represents the longitudinal compressive strength, *X_t_* is longitudinal tensile strength, *Y_c_* stands for the transverse compressive strength, *Y_t_* is transverse tensile strength, *S*_12_ denotes as the shear strength. The material parameters of different composite panels are listed in Table 3. The modulus, Poisson’s ratio, strength, etc. are obtained by quasi-static experiments according to ASTM standard, of which the strength of 2DTBC are referred to [32]. 

Before the satisfaction of failure criterion, the stress–strain responses along the fiber direction (1–direction), transverse direction (2-direction) and shear direction (1–2 direction) are illustrated by the following equations:

(1) $$\varepsilon_{1}=\frac{1}{E_{1}}\left(\sigma_{1}-\nu_{12}\sigma_{2}\right)$$

(2) $$\varepsilon_{2}=\frac{1}{E_{2}}\left(\sigma_{2}-\nu_{21}\sigma_{1}\right)$$

(3) $$2\varepsilon_{12}=\frac{1}{G_{12}}\left(\tau_{12}+\alpha \tau_{12}^{3}\right)$$

where *ε* indicates the strain, *σ* and *τ* stand for the normal stress and shear stress, respectively. *E* and *G* represent Young’s modulus and shear modulus, respectively. In addition, the coefficient *α* is a weighing factor describing the nonlinear shear stress–strain response [33].

For the prediction of fiber tensile failure, the influence of shear stress to fiber–tensile failure can be defined explicitly through calibrating the weighing factor *β*. The maximum stress failure criterion is implemented when *β* = 0 and the Hashin failure criterion [34] is achieved while setting *β* = 1. Furthermore, strength reduction parameters *FBRT* and *YCFAC* are introduced to model the effect of matrix damage on fiber failure, based on the following two equations [33,35]:

(4) $$X_{t}=X_{t}^{*}\times FBRT$$

(5) $$X_{c}=Y_{c}^{*}\times YCFAC$$

## 4. Results and Discussion

Finite element models were developed for each type of composite specimen to investigate their different failure behavior under ballistic impact loads. The models were first validated against experimental results based on their impact threshold and failure patterns. Then, sysmetical numerical studies were conducted to assess the damage tolerance of each type of composite panel.

### 4.1. Model Validation

**Figure 3:**
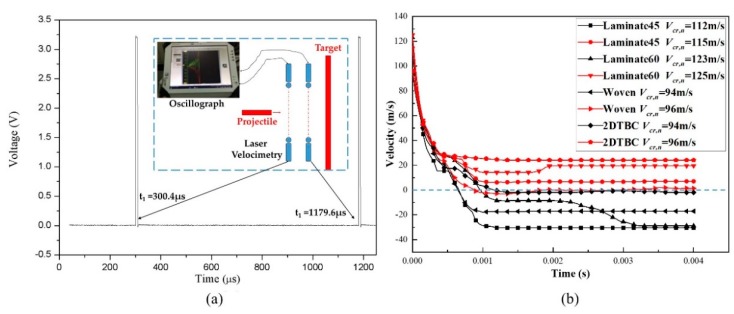
(**a**) Laser velocimetry test system in experiments, (**b**) numerical simulation results of the projectile velocity profiles for the impact tests against different composite panels.

The velocity of the projectile was measured by laser velocimetry, where two parallel laser beams were arranged in front of the target. The laser velocimetry consisted of a laser generator and a receiving device with a constant distance of *d*. The laser receiving device is connected to the high-frequency data acquisition and is displayed as an incident signal on the oscilloscope. When the projectile passes through the laser beam, the laser beam is blocked by the flying missile, and the signal collected on the waveform will produce a sharp wave, as shown in Figure 3a. The time interval between time *t*_1_ and *t*_2_ as recorded by the oscilloscope, corresponds to the period the projectile flying through the two laser beams. Thus, the initial impact velocity of the projectile can be calculated as

(6) $$V_{e}=\frac{d}{t_{2}-t_{1}},$$

The FE models were first validated by comparing the numerical predicted critical penetrating velocity for each kind of composite panel against the experimental results. The critical penetrating velocity is defined as a range, of which the lower bound is the maximum speed that the projectile cannot penetrate the composite panel and the upper bound is the minimum speed that the panel is penetrated. Table 4 summarizes the experimental and numerical results of the critical penetration velocity range for the four different panels. The results illustrate that the proposed numerical model can predict the impact resistance of woven and angle-ply laminates in a good manner. However, the numerically predicted results for the 2DTBC panel are a little bit lower due to the ignorance of the complicated braided architecture.

Comparing the different composite materials, we noticed that the penetration resistance of the angle-ply laminates is obviously better than the textile composites (including both the woven and 2DTBC panels), although the stacking sequence of the laminate is designed to be consistent with the structure of the woven fabric and 2DTBC laminates. The numerically predicted velocity histories of the projectile under the critical impact velocities for each panel are plotted in Figure 3b. As shown in Figure 3b, the velocity of the projectile declines drastically at the initial stage and stabilizes in its residual velocity after the impact. The negative value of residual velocity indicates that the projectile is rebounded by the composite panel, otherwise, the panel is penetrated by the projectile. 

**Figure 4:**
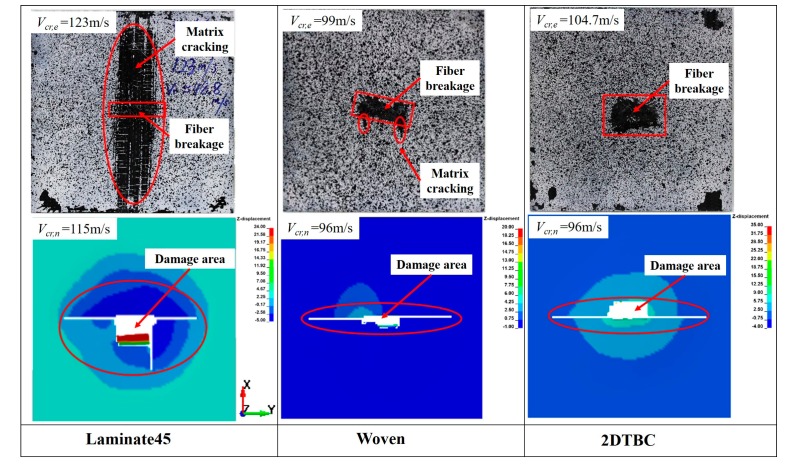
Impact failure patterns obtained by experiments and numerical simulations.

Figure 4 compares the simulation and experimental results of impact failure patterns for Laminate 45, woven, and 2DTBC at the impact velocities *V_cr,n_* and *V_cr,e_*. As from both experiments and numerical simulation, Laminate 45 and Laminate 60 specimens show similar impact failure patterns. Thus, we list only the test results of Laminate45. The experimental results show that matrix cracking and delamination damage is very significant for the laminate composite, propagating along the fiber direction and distributing across almost the whole un-constrained area. The damage area of the woven and 2DTBC panels is relatively small, showing a concentrated fiber–shear breakage zone and a much smaller area of matrix cracking. Numerically, Laminate45 shows a relatively larger damage area and a much larger out-of-plane deformation compared to those of the woven and 2DTBC panels, which is consistent with the experimental observations. The results also indicate that the current homogeneous models are inadequate in predicting the complicated failure behavior of composite structures. However, considering the computational efficiency and the good prediction of impact threshold, the current models provide good knowledge for the understanding of the impact resistance of different composite panels.

### 4.2. Damage Progression Behavior

**Figure 5:**
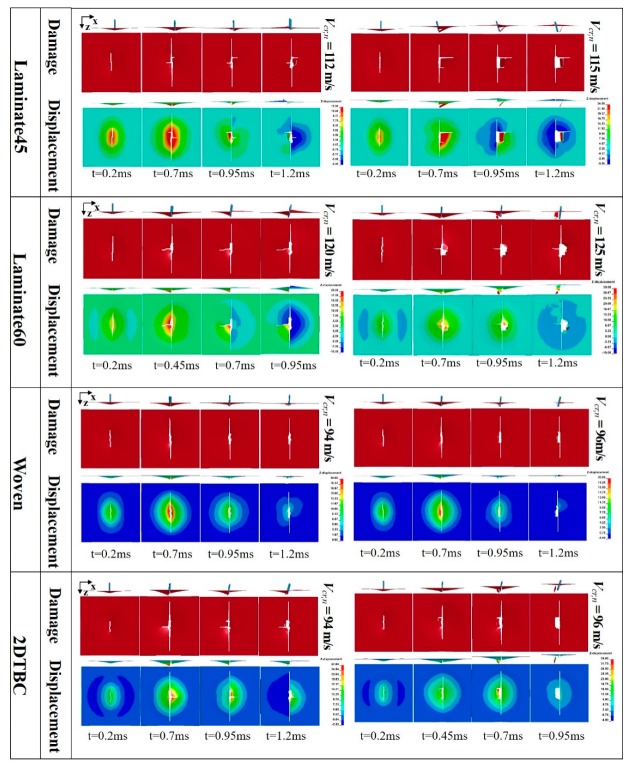
Numerical predicted impact damage patterns and displacement distributions for Laminate45, Laminate60, woven, and 2DTBC composite panels at different stages.

The validated models are then applied to investigate the impact damage behavior of the four different composite panels, through comparing the damage patterns and displacement contours during the impact process. As shown in Figure 3, the impact velocity of the projectile is selected as the critical penetration velocity (*V_cr,n_*) for comparison purposes. For all cases, there is a principal crack initiating from the center of the plate and propagating along the transverse direction that is parallel with the width of projectile. As from the damage patterns, the damage area (or crack length) along the axial direction (*x*-direction) is more serious in Laminate45 and Laminate60 than that of the woven and 2DTBC panels. The crack along the *x*-direction for the woven and 2DTBC composite panels is almost negligible. This is also consistent with the deformation contour plots. As from Figure 5, the displacement distribution for the textile (woven and 2DTBC) composite panels spread more widely than that of the laminates, with also a larger maximum out-of-plane displacement. While for the laminates the damage is more concentrated, resulting a larger damage area but a higher energy absorption capacity. The balanced in-plane stiffness properties of the textile composite panels result in a larger deformation area and a relatively smaller damage zone, which to a certain extent can facilitate the enhancement of the resultant strength of impact-damaged panels.

The numerical simulated damage patterns of 2DTBC are similar with those reported in [6,7], which also used a highly efficient homogenized model to simulate the impact performance of a two-dimensional triaxial braided composite casing. While the previous studies [6] were concerned with the effect of projectile shape, this study analyzed more extensively the damage morphology of composites with different architecture.

### 4.3. Impact Damage Tolerance of Composite Panels

The impact attitude of the projectile has a great influence on the damage or failure modes of composite structures. Good control of the impact attitude and velocity of the projectile is very important for a successful high-velocity impact test. Additionally, an examination of the impact attitude is necessary for the structural design. However, in realistic application, the composite structure may suffer more complicated impact loads than in experiments, which makes it necessary to study the impact damage tolerance of the structure under different impact conditions. 

**Figure 6:**
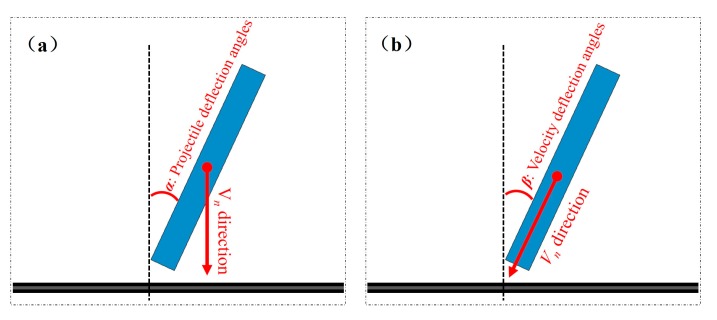
Sketch map of the different impact attitudes at *V_n_*, (numerical impact velocity of the projectile), (**a**) projectile deflection angle *α*, (**b**) velocity deflection angle *β*.

The validated FE model is applied to study the effects of impact attitude on the failure behavior of composite panels. The attitude of the projectile is difficult to capture during the impact process, so the complex attitude of the projectile is decomposed into a simple attitude for analysis, thereby obtaining the sensitivity of impact resistance against the complex impact attitude. Two typical impact conditions are parametrically studied, as shown in Figure 6. Case 1: deflection angle of the projectile changes but the impact direction is perpendicular to the panel; and Case 2: the impact direction is parallel with the projectile deflection angle. As from Figure 6, the deflection angle of the projectile is denoted in *α* (projectile deflection angle), and the angle between the impact direction and the normal direction of the target panel is defined as *β* (velocity deflection angle). The damage modes under different impact conditions with varying angles (*α* = 10°, 20°, 30°, 45°; and *β* = 10°, 20°, 30°, 45°) are numerically examined, respectively.

#### 4.3.1. Effect of Projectile Deflection Angles on Impact Damage

**Figure 7:**
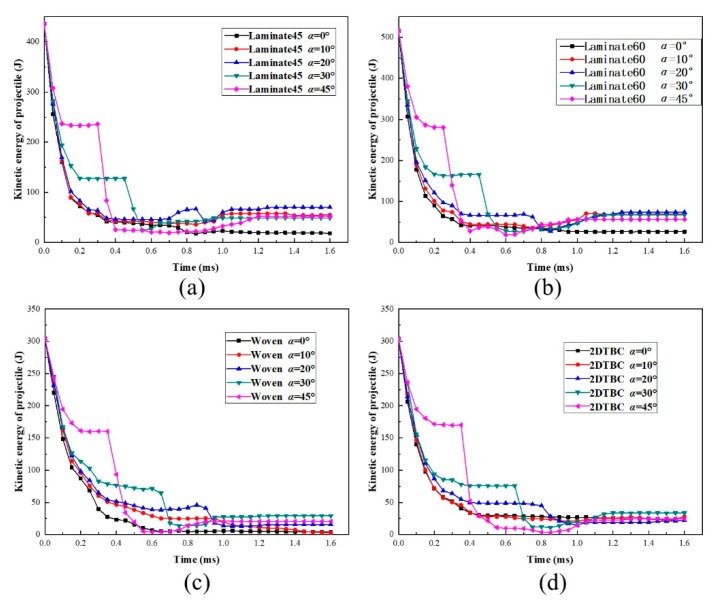
Evolution of kinetic energy of projectile for composite panels against the change of *α*: (**a**) Laminate45 at *V_cr,n_*=115 m/s, (**b**) Laminate60 at *V_cr,n_*=125 m/s, (**c**) woven panel at *V_cr,n_*=96 m/s, (d) 2DTBC panel at *V_cr,n_*=96 m/s.

The different projectile impact attitudes were set for the validated models, and the obtained results were analyzed for the energy change process during the impact process. Figure 7 compares the numerically predicted energy profile results for the four different kinds of composite panels, under critical impact velocity with various projectile deflection angles *α*. Obviously, the results of all composite panels conform to the law that multi-stage energy transmission behavior presents at a large projectile deflection angle (*α* > 20°) and the duration time of first-stage transformation decreases with the increase in projectile deflection angles. In addition, the kinetic energy of the secondary impact increases remarkably with the increase of projectile deflection angle *α*.

**Figure 8:**
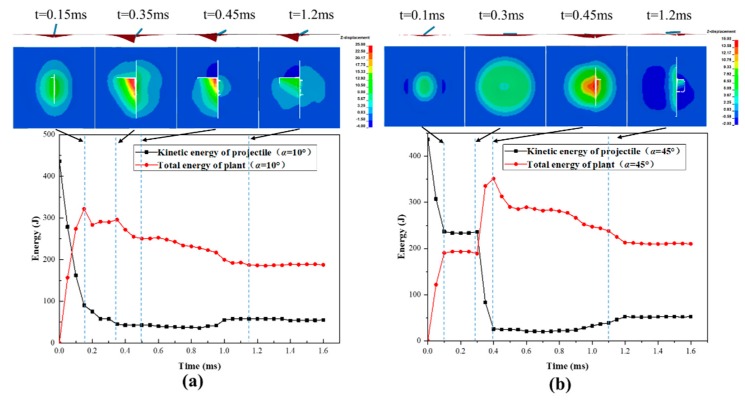
Energy evolution process and impact damage process for Laminate45 under impact *V_cr,n_* = 115 m/s with two different projectile deflection angles of *α* = 10° (**a**) and *α* = 45° (**b**).

To further understand the process, the energy conversion and transmission process is further analyzed together with the impact failure images, taking Laminate45 as an example, as shown in Figure 8a,b. According to the kinetic energy profiles of the panel and the projectile, the whole impact process can be divided into four stages. The first stage corresponds to the initial contact between the panel and the projectile, during which the kinetic energy of the projectile decreases rapidly and transmits into the deformation of the composite panel. Following this, the energy curves flatten, where the projectile starts to rotate around the impact point due to the bias between the longitudinal axis and the velocity direction of the projectile. In this stage, the projectile penetrates the panel for the case *α* = 10° while results in a secondary impact for the case *α* = 45°.

The impact continues during the third stage, where the rotating and secondary impact by the tail of the projectile causes continuous damage propagation, resulting in further energy declining of the projectile. It is also found that energy transmission is more significant for the case *α* = 45°, where the contact area is much larger during the secondary impact process. The final stage corresponds to the penetration or rebounding process and usually shows relatively minor energy transmission. Comparing the two cases, we found that when the projectile deflection angles are small the kinetic energy loss is the most at the first stage, while when the projectile deflection angles are big the secondary impact causes the most loss of kinetic energy. This suggests that the projectile deflection angles may change impact failure behavior and energy transmission mechanisms.

#### 4.3.2. Effect of Velocity Deflection Angles on Impact Damage

**Figure 9:**
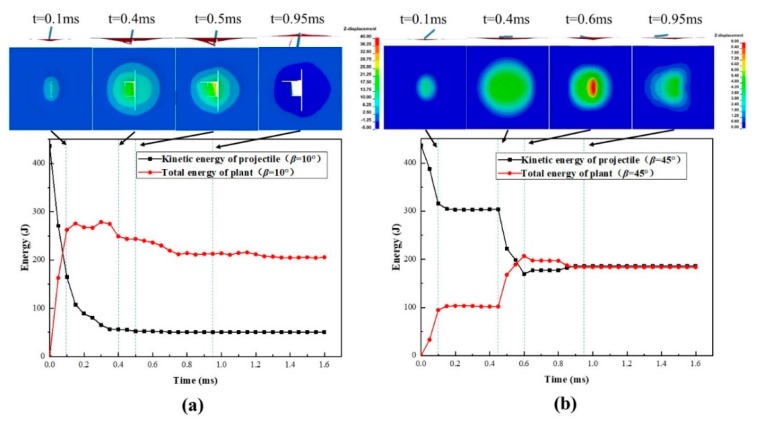
Energy evolution process and impact damage process for Laminate60 under impact *V_cr,n_*=125m/s with two different velocity deflection angles: (**a**) *β* = 10° and (**b**) *β* = 45°.

When the impact direction varies with the velocity deflection angles of the projectile in a small angle (*β* = 10°), the projectile will penetrate the panel without any rotation, so there is no secondary impact damage during the penetration, as is shown in Figure 9a. However, with the continuous increase of *β*, the panel is hard to penetrate by the projectile, and the composite panel will be subject to the secondary impact damage, as shown in Figure 9b. In general, the out-of-plane deformation caused by the secondary impact is much serious that the first impact. Compared with the impact of projectile deflection angles *α* = 45° in Figure 8b, the composite panel shown in Figure 9b has no explicit damage after the secondary impact, and the residual energy of the projectile at *β* = 45° is significantly larger than that at *α* = 45°, and the damaged area is also much smaller than that of *α* = 45°.

**Figure 10:**
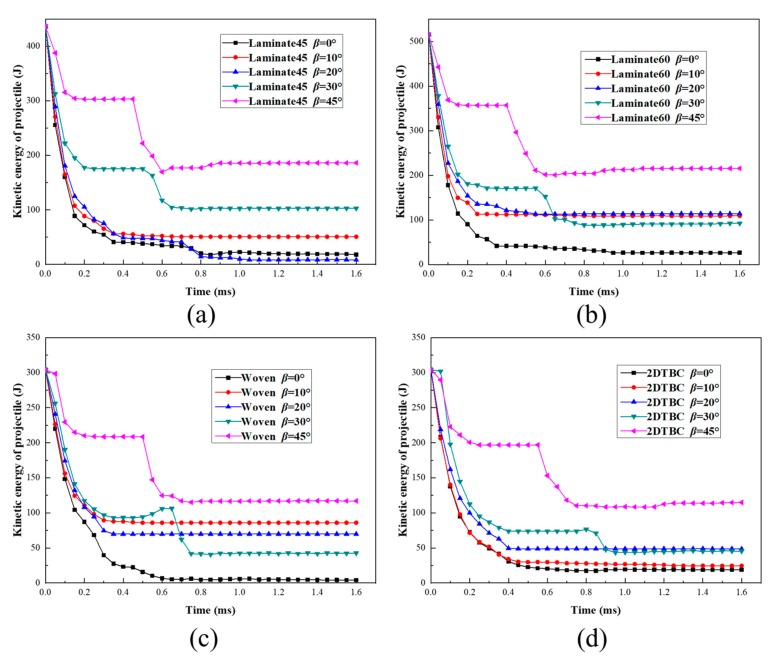
Effect of deflection angle on the kinetic energy profile of different composite panels, under critical impact velocities: (**a**) Laminate45 at *V_cr,n_* = 115 m/s, (**b**) Laminate60 at *V_cr,n_* = 125 m/s, (**c**) woven panel at *V_cr,n_* = 115 m/s, (**d**) 2DTBC panel at *V_cr,n_* = 115 m/s.

The value of *β* is taken as 0°, 10°, 20°, 30°, and 45°, respectively, and the kinetic energy of the projectile is shown in Figure 10. In 20° < *β* < 45°, the residual energy of the projectile after the second impact is increasing with the increases in *β*. At different angles, the residual energy of the projectile is concentrated in a small range at the angles. On the contrary, the residual energy value of the projectile is relatively larger at different values of *β*.

#### 4.3.3. Comparison Studies on the Effect of α and β

**Figure 11:**
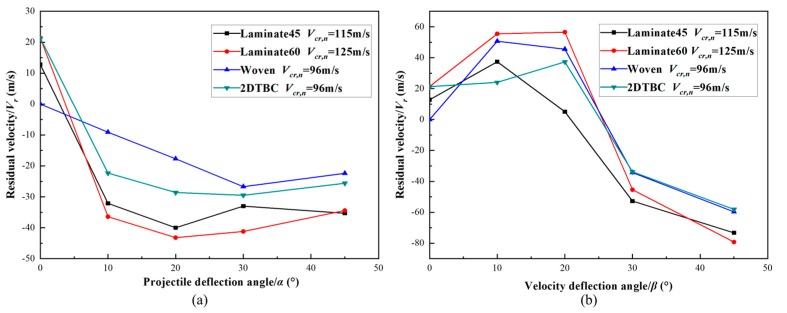
The relationship between deflection angles and residual velocity. (**a**) Relationship of projectile deflection angles *α* and residual velocity, (**b**) relationship of velocity deflection angles *β* and projectile residual velocity.

Figure 11a plots the relationships of projectile deflection angles *α* and residual velocity *V_r_* of the projectile for the composite panels under critical penetration velocity. As from Figure 11a, there is a sharp decreasing of residual velocity against the increase of projectile deflection angles *α*, due to the dissipation of kinetic energy for rotation motion of the projectile. Further increasing the projectile deflection angles, there is little variation for the residual velocity as the impact behavior and energy transmission behavior is similar for those cases. For the four different kinds of composite panels, the evolution tendencies for the relationship between the residual velocity of the projectile (*V_r_*) and deflection angles are similar, which suggests that the penetration probability of target plate is reduced when the projectile deflection angles increases.

The relationships between the velocity deflection angle *β* and residual velocity of the projectile for different composite panels are described in Figure 11b. The projectile penetrates the panel while the angles *β* is smaller than 20° but is rebounded by the panel once the angle goes through a threshold. As from these results, the residual velocity reaches the maximum value when *β* equals 20°, where for the 0~20° region, the increase of deflection angle generates more damage due to the stress/strain concentration. Followed by which the damage area of the panel is reduced with the increase of velocity deflection angle, as the velocity component along the tangential direction of the panel get decreased.

The effect of projectile deflection *β* on residual velocity has also been studied by Chen et al. [36], which concerned the high-speed impact performance of a lithium-ion battery using a homogenized anisotropic model and identified the presence of a critical deflection angle for a maximum residual velocity *V_r_*. Comparing Figure 11a and b, for the small deflection angle range, the damage induced by the impact with *β* is more serious than that with *α*. For the rest angle range, the damage caused by impact with *β* is less than with *α*. Overall, the most serious impact damage of the composite panels corresponds to impact with velocity deflection angles *β* in the range of 10°–20°. 

### 4.4. Impact Resistance of Different Composite Panels

**Figure 12:**
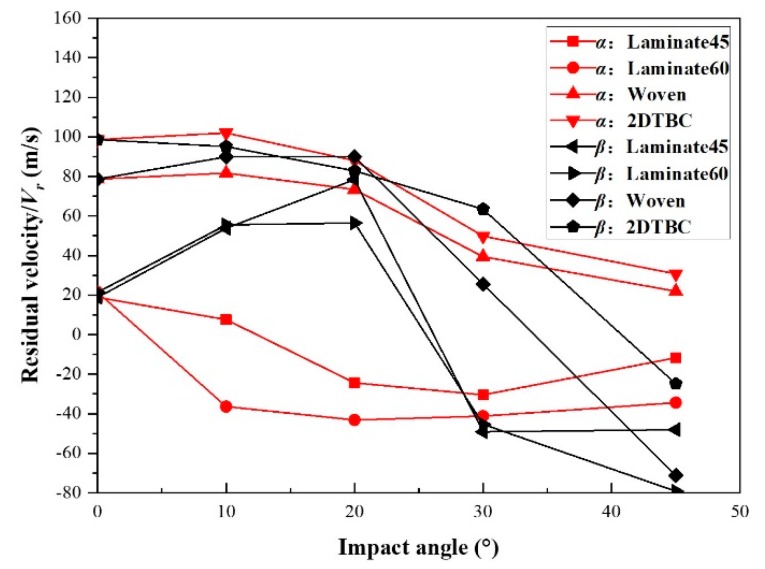
The relationship between deflection angle α, *β*, and projectile residual velocity for composites panels impacted under *V_n_* = 125 m/s.

To further understand the impact resistance of different types of composite panels, numerical simulations were conducted for them individually, for the same impact condition with *V_n_* =125 m/s (*V_n_* takes the value of the maximum *V_cr,n_* of the four kinds of composite panels, i.e., *V_n_* = max(*V_cr,n_*)). Figure 12 shows the results for the relationships between residual velocity and deflection angle *α* and *β*. 

For Laminate45, although the impact velocity is higher than the critical velocity, the curvature of residual velocity against the deflection angle still follows the tendencies shown in Figure 11a,b. When the deflection angles (*α* and *β*) is less than 40°, the residual velocity *V_r_* of laminate panels (Laminate45 and Laminate60) are lower than that of textile composites (woven and 2DTBC). For the same impact condition, the penetration resistance of laminates is better than that of the textile composites. This indicates that the laminates hold better energy-absorption capability under ballistic impact than the textile composite panels, due to the larger and more serious failure behavior.

For the textile composites, under an impact velocity much higher than the critical velocity, the residual velocity *V_r_* does not change much when *α* and *β* are lower than 20°. This suggests that the residual velocity *V_r_* of textile composites is less sensitive to the change of the impact attitude.

As the angle increases, at 20° < *α*, *β* < 40°, the residual velocity *V_r_* of the woven and 2DTBC panels starts to show sensitivity to the change of impact attitude and decreases drastically against the increase of deflection angle. Also, the same as in Figure 11, the residual velocity shows a more obvious sensitivity to velocity deflection angle *β*. As *β* increases from 30° to 40°, the residual velocity decreases from positive to negative, indicating a complete change of tolerance capability. While the residual velocity *V_r_* of woven and 2DTBC panels is decreasing moderately with the increase of projectile deflection angle *α*, showing a reduced sensitivity. Overall, it is found that when the angles *α* and *β* are larger than 20°, the damage tolerance for the composite panels is substantially unchanged against the change of projectile deflection angle *α*, while the impact resistance changes a lot against the change of velocity deflection angle *β*. Most of the existing studies deal with the effect of projectile shapes and material properties on the damage behavior of composite plates [2,3,4,5,6,7,8,9]. This study provides additional understanding on the effect of architecture type and impact attitudes on the impact resistance of composite panels, which could be useful for the design of composite protective structures.

## 5. Conclusions

In this paper, a highly computational efficiency finite element model was built to solve the difficult convergence and nonlinear problems for the impact of laminate and textile composites. The numerical models were validated by comparing them with experimental results. Systematical numerical case studies were conducted to understand the variation of damage tolerance for different types of composites under various impact conditions. The failure behavior and impact resistance of three different types of composite panels were comprehensively analyzed, which provides guidance for the anti-impact design and optimization of composite aero-engine structures. The study achieves the following conclusions:

1. The finite element model, using shell element with multiple integration points along the thickness, provides good accuracy in predicting the impact threshold of composites panels, and reasonable prediction of failure behavior.
2. The laminated composites show better resistance against high-speed ballistic impact, but more serious deformation and larger damage areas, than those of textile composites.
3. The impact attitude of the projectile affects the penetrating capability of the projectile. The composite panels are more likely to be penetrated when the velocity deflection angle is 10° < *β* < 20°.
4. The tolerance capability of the composite panels changes moderately when the deflection angles are smaller than 20°, but shows a more obvious sensitivity when the deflection angles go beyond 20°.
5. The impact resistance of the composite panels is more sensitive to velocity deflection angle *β* than against projectile deflection angle *α*. The textile composites show moderate sensitivity to the deflection angle than the laminate composites.

## Figures and Tables

**Table 1:** Geometry and layup information of the studied composite panel specimens.

	Laminate45	Laminate60	Woven	2DTBC
Lay-up design	[0/90(45/-45/0/90)_2_/90/0]_S_	[0/60-60/-60/60/0]_2S_	[0/(45/0)_4_/0]_S_	[0]_8_
Thickness(mm)	4.45	4.45	3.8	4.5
Layers	24	24	20	8
Number of integration points	72	72	60	24
Fiber volume ratio	61.2%	61.2%	59.6%	55%

**Table 2:** Chang–Chang failure criterion and stiffness degradation method used in this work.

Failure Model	Failure Criterion	Stiffness Degradation Method
Fiber tensile failure mode	$\sigma_{11}\geq 0:2e_{f}^{2}={\left(\frac{\sigma_{11}}{X_{t}}\right)}^{2}+\beta \left(\frac{\sigma_{12}}{S_{12}}\right)\geq 1$	$\begin{array}{l} E_{1}=E_{2}=G_{12}=0\\ \nu_{12}=\nu_{21}=0 \end{array}$
Fiber compressive failure mode	$\sigma_{11}<0:e_{c}^{2}={\left(\frac{\sigma_{11}}{X_{c}}\right)}^{2}\geq 1$	$\begin{array}{l} E_{1}=0\\ \nu_{12}=\nu_{21}=0 \end{array}$
Matrix tensile failure mode	$\sigma_{22}\geq 0:e_{m}^{2}={\left(\frac{\sigma_{22}}{Y_{t}}\right)}^{2}+{\left(\frac{\sigma_{12}}{S_{12}}\right)}^{2}\geq 1$	$\begin{array}{l} E_{2}=G_{12}=0\\ \nu_{21}=0 \end{array}$
Matrix compressive failure mode	$\begin{array}{l} \sigma_{22}<0:e_{d}^{2}={\left(\frac{\sigma_{22}}{2S_{12}}\right)}^{2}+{\left(\frac{\sigma_{12}}{S_{12}}\right)}^{2}+\\ \left[{\left(\frac{Y_{c}}{2S_{12}}\right)}^{2}-1\right]\frac{\sigma_{22}}{Y_{c}}\geq 1 \end{array}$	$\begin{array}{l} E_{2}=G_{12}=0\\ \nu_{12}=\nu_{21}=0 \end{array}$

**Table 3:** Model parameters of MAT54 for the three types of composites

Variable	Laminate	Woven	2DTBC
RO: Mass density/ton × mm^−3^	1.68 × 10^−9^	1.36 × 10^−9^	1.65 × 10^−9^
EA: Young’s modulus-longitudinal direction/MPa	139,000	55,000	44,000
EB: Young’s modulus-transverse direction/MPa	6655	55,000	44,000
EC: Young’s modulus-normal direction/MPa	6655	7100	7000
PRBA: Poisson’s ratio *ν_ba_* = *ν*_12_	0.0138	0.3500	0.3000
PRCA: Poisson’s ratio *ν_ca_* = *ν*_31_	0.0138	0.0331	0.0477
PRCB: Poisson’s ratio *ν_cb_* = *ν*_32_	0.4450	0.0294	0.0461
GAB: shear modulus *G_ab_*/MPa	3346	5100	4000
GBC: shear modulus *G_bc_*/MPa	3346	5100	4000
GCA: shear modulus *G_ca_*/MPa	2302	4100	3200
DFAILT: Max strain for fiber tension	0.023	0.023	0.023
DFAILC: Max strain for fiber compression	−0.022	−0.022	−0.022
DFAILM: Max strain for matrix straining in tension and compression	0.042	0.042	0.042
DFAILS: Max shear strain	0.032	0.032	0.032
ALPH: Shear stress non-linear term (ALPH = *α*) in Equation (3)	0.85	0.85	0.85
FBRT: Softening factor for fiber tensile strength after matrix failure	0.59	0.59	0.59
YCFAC: Softening factor for fiber compressive strength after matrix failure	1.2	1.2	1.2
BETA: Shear stress weighing factor in tensile fiber mode	0.5	0.5	0.5
XT: Longitudinal tensile strength *X_t_*/MPa	2961	1051	700
XC: Longitudinal compressive strength *X_c_*/MPa	2665	393	390
YT: Transverse tensile strength *Y_t_*/MPa	64	1051	540
YC: Transverse compressive strength *Y_c_*/MPa	127	393	302
SC: Shear strength *S*_12_/MPa	63	120	257 [32]

**Table 4:** Comparison of experimental measured (*V_cr,e_*) and numerical predicted (*V_cr,n_*) critical penetration velocities (m/s).

Specimen	Experimentally Measured Velocity Threshold	Numerical Predicted Velocity Threshold
Laminate45	117 < *V_cr,e_* < 123	112 <*V_cr,n_* < 115
Laminate60	*V_cr,e_* < 125	123 <*V_cr,n_* < 125
Woven	94 <*V_cr,e_* < 99	94 <*V_cr,n_* < 96
2DTBC	100.6 < *V_cr,e_* < 104.7	94 <*V_cr,n_* < 96
